# The Ethnoveterinary Study of Medicinal Plants Utilized in Treating Animal Diseases in Ensaro District, North Shewa Zone of Amhara Regional State in Ethiopia

**DOI:** 10.1155/tswj/3038829

**Published:** 2025-07-09

**Authors:** Mikias Teshome, Tamene Yohannes

**Affiliations:** Crop & Horticulture Biodiversity Research, Ethiopian Biodiversity Institute, Addis Ababa, Ethiopia

**Keywords:** Ensaro woreda, ethnoveterinary, traditional medicine

## Abstract

An ethnobotanical study was conducted to document the plant-based indigenous knowledge of the people on the utilization of these medicinal plant resources in Ensaro District, North Shewa Zone, Ethiopia. A total of 100 informants were sampled from four study sites, and a variety of ethnobotanical methods were applied, including semistructured interviews, field observations, and direct matrix rankings. The vast source of traditional healing knowledge of plant species conveyed from one generation to the next by word of mouth was in a family member. Totally 47 plant species were identified from the study site. These 47 medicinal plants belong to 44 genera and 31 families. Of these, 21 species are used for the treatment of livestock ailments only, and 26 species are reported for the treatment of both human and livestock ailments. These medicinal plants are used to treat about nine types of animal ailments and eight types of animal and human ailments. Family Solanaceae, Lamiaceae, and Asteraceae were represented by the highest number of five medicinal plant species, followed by the Fabaceae with three, Rutaceae two, and the remaining family representing one species. Of the total medicinal plant species, 24 species (51%) were shrubs, 16 species (34%) were herbs, and 2 species (4%) were trees, whereas 4 species (9%) were climbers. Most of them have medicinal properties in their leaf, bark, root, stem, flower, seeds, and fruits. Medicine from these plant parts is prepared in fresh, dried, and both fresh and dried states. Data showed that retained placenta had the highest informant consensus factor (ICF) value (1.00), followed by anthrax and eye infection (0.9), diarrhea (0.896), rabies (0.888), leech infestation (0.808), and snake poisoning (0.750). The highest fidelity level (FL) values were obtained for the plants *Sideroxylon oxyacanthum* treating leech infestation, *Inula confertiflora* used to treat eye infection, and *Nicotiana tabacum* also for leech infestation. Therefore, further phytochemical investigations need to be conducted on the above-listed plants with the highest FL values, which may indicate their higher potential against the respective ailments. Due to high population growth, the expansion of urban areas, and the need for more farming lands, there are significant challenges in conserving ethnoveterinary medicinal plants. The existing conservation efforts undertaken by the local community are insufficient to address the loss of plant species from their natural habitats. Therefore, it is imperative to implement both *in situ* and *ex situ* conservation strategies based on the nature of medicinal plants.

## 1. Introduction

Ethiopia is one of the African countries that have the largest livestock population, despite having a substantial livestock population compared to other African nations; Ethiopia's livestock sector has not made a significant contribution to the country's economy. This can be attributed largely to the prevalence of diseases that hinder its growth. Additionally, the development and distribution of modern veterinary medicine in the country are inadequate, particularly in livestock production areas. Ethiopian farmers and pastoralists heavily depend on traditional knowledge, practices, and locally available resources to control and manage livestock diseases [1]. According to [2], approximately 80% of Ethiopia's population relies on traditional medicine, with 95% of these treatments derived from medicinal plants. The plant is the most essential to human well-being in providing basic human needs. Human beings started using plants for disease control and prevention since time immemorial. According to [3], ethnobotany is a wide term referring to the study of people's classification, management, and use of plants. It is defined as local people's interaction with plants, and how they classify, manage, and use plants available around. Early humans acquired knowledge on the utilization of plants for disease prevention and curative purposes through many years of experience, careful observations, and trial and error experiments [3], and it is estimated that about 75%–90% of the rural population in the world excluding Western countries depend on traditional medicines [4].

In most developing counters, where poultry health services are scarce and not well developed and animal production is located far from these clinics. Livestock disease is still a major factor in low cattle productivity. This leads to an increasing gap between the supply and demand for livestock and related products in numerous developing nations. A significant portion of farmers still now depend on traditional medicine, especially herbal remedies, to maintain the health and productivity of their livestock over many decades [5]. Ethnoveterinary practices are more common in developing countries and using homemade remedies made from various plant materials because traditional ethnoveterinary remedies provide a cheaper and more accessible comparing with modern drugs [6]. In rural areas where access to veterinary services and facilities is limited, traditional medicinal plants are often the primary option for livestock keepers to treat various ailments [7]. Although traditional veterinary practices are crucial for maintaining the health of livestock, they have not been well-documented in Ethiopia [8]. Raising awareness among animal keepers about the advantages of utilizing plant-based remedies in ethnoveterinary medicine is crucial for effective livestock management. Further to ensure successful livestock production, it is important to have a thorough understanding and documentation of farmers' knowledge, attitudes, and practices regarding the causes, treatments, prevention, and control of various ailments [9].

Natural vegetation plays a crucial role in ethnoveterinary practices as a source of medicinal plants and resources for animal healthcare. In Ethiopia, where traditional knowledge and practices are deeply rooted in local communities, natural vegetation serves as a rich reservoir of plant species with medicinal properties that have been used for generations to treat various livestock ailments. Thus, efforts needed to conserve biodiversity and traditional knowledge of medicinal plants are essential for ensuring the continued availability and effectiveness of ethnoveterinary practices in Ethiopia and beyond. The rapid disappearance of medicinal plant species is a result of various factors, including environmental degradation, agricultural expansion, deforestation, and overharvesting. The rise in human and livestock populations in Ethiopia has intensified a particular issue, leading to the accelerated loss of biological diversity and indigenous knowledge.

The ethnoveterinary medicinal plant knowledge in Ethiopia, like other forms of traditional knowledge, lacks proper documentation [10]. This rich knowledge held by traditional medical practitioners is at risk of being lost forever when these practitioners pass away without adequately sharing their knowledge. Furthermore, the younger generation shows hesitancy towards adopting the conventional way of life, thus posing a greater risk to the preservation of this invaluable knowledge [8, 11, 12]. The present study focused on identifying medicinal plants used to treat livestock ailments, plant habits, diseases treated, ways of knowledge acquired, methods of preparation, routes of administration, ingredients added during the preparation of medicine, and conservation practices of the local communities. The study on animal disease remedies in Ethiopia is limited despite the country's abundant plant diversity, multilingualism, and high livestock population in Africa. Therefore, this study is necessary to collect and document the traditional use of medicinal plants used to treat livestock ailments available in Ensaro District, North Shewa Zone of Amhara Region, Ethiopia. This research could be helpful as source information for the researcher who wants to further studies on medicinal plants in Ensaro District and for future pharmacological and phytochemical studies. Therefore, this research paper documents ethnoveterinary medicinal plants used in the study districts, Ensaro District of Northern Ethiopia.

## 2. Materials and Methods

### 2.1. Description of the Study Area

The current investigations carried out in Ensaro woreda, which is located in the North Showa Zone of Amhara Regional State in Ethiopia. This is located between 9° 35⁣′–9° 55⁣′N and 38° 50⁣′–39° 5⁣′E, with an average elevation of 2435 m above sea level. The district consists of one urban and 13 rural kebeles. Lemmi town is the headquarters of the woreda and is located 130 km northwest of Addis Abeba. According to the 2007 national census performed by Ethiopia's Central Statistical Agency (CSA), the woreda has a total population of 58,203, of which 29,888 were male and 28,315 were female; 3164 (5.44%) were urban people. The woreda is bounded by the Oromia Region to the south and west, the Jemma River to the north, which separates it from Merhabiete woreda, Moretnajiru to the northeast, and Siyadebrina Wayu woreda to the east.

**Figure 1:**
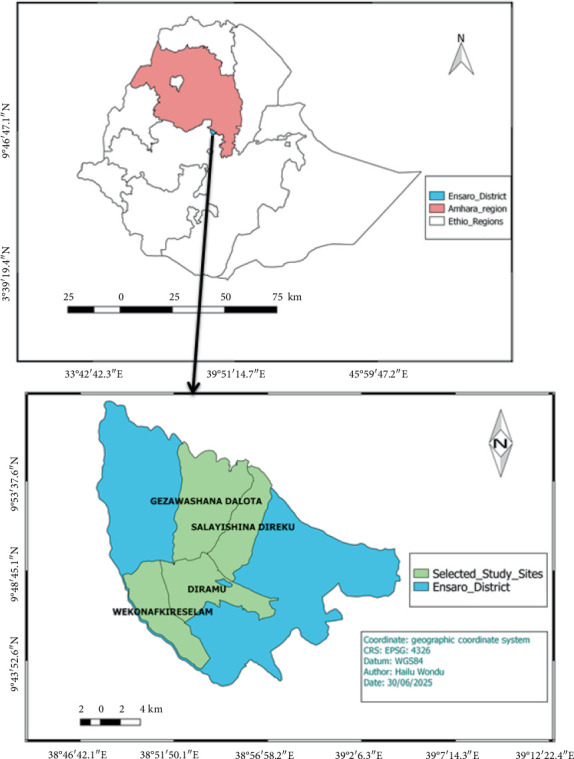
Study area map.

A variety of sampling strategies were used to find the most appropriate sample region. Ensaro woreda was chosen specifically because of its natural forest, low infrastructure, and road accessibility. A preliminary survey was undertaken to evaluate the research site and assist in the selection of certain kebeles (small administrative divisions) for the study. Following the reconnaissance survey, four kebeles were selected based on a variety of criteria (Figure 1), including agroecology, vegetation cover, and the presence of traditional practitioners. These chosen kebeles are located in various locations within the research region.

### 2.2. Climate

The climate plays a crucial role in determining the distribution of animals and influencing settlement patterns. It influences the lifestyle of people and shapes the types of soil, plants, and animals present in a specific region. Since Ethiopia is primarily agricultural, rainfall and temperatures have a significant impact, particularly in the study area. Meteorological data spanning 20 years (1998–2018) from the Addis Ababa National Meteorology Service Agency (recorded at Lemi station) reveal that the rainfall follows a Unimodal pattern, with peak rainfall occurring between June and August and lower rainfall levels from March to May. The dry season typically lasts from September to February. Over the two-decade period, the mean annual rainfall in the study area was 1224 mm, with the lowest mean annual temperature recorded at 8.8°C and the highest at 20°C.

### 2.3. Informant Sampling and Ethnobotanical Data Collection

The study involved selecting 100 informants between the ages of 22 and 82. From these 100 informants, 80 were chosen randomly, while the remaining 20 were purposefully selected as key informants. The selection of key informants was based on information and recommendations from local healers, kebeles administrators, and kebeles developmental agents. This was done to gather detailed information about the use of medicinal plants. The collection of ethnoveterinary data took place between December and March 2019. The data collection process involved close interaction with the informants using semistructured questions. These questions were prepared beforehand in English and then translated into Amharic, the language spoken by the inhabitants, for the interview administration. The interviews were conducted based on a checklist, with additional topics being explored based on the responses given by the informants. All of the interviews were held in Amharic. In a more structured interview, the healers were asked about plants, the use(s) and method of preparation of plants, and the route of administration of the herbal preparations. Based on ethnobotanical information provided by informants, collected specimens during guided field walks were pressed, numbered, dried, and given vernacular names on each sheet and dried for identification. Then, the plants were pressed, dried, and taken to the Ethiopian Biodiversity Institute (EBI) for identification.

### 2.4. Analysis of Ethnobotanical Data

Data were analyzed following survey and analytical tools for ethnobotanical methods recommended by Martin, Banerjee, and Fakchich and Elachouri [3, 13, 14]. The ethnobotanical data were analyzed using quantitative and qualitative methods of data analysis. Descriptive statistics such as percentage, frequency distribution, and graphs used to analyze the data collected through semistructured, open-ended, and some close-ended questions.

#### 2.4.1. Informant Consensus Factor (ICF)

The level of agreement among informants in the district regarding the use of medicinal plants to treat a specific ailment category was determined by calculating ICF values. These values can be useful in selecting medicinal plants for further phytochemical and pharmacological studies. The ICF values were computed using the formula ICF = (nur − nt)/(nur − 1), where nur represents the number of user reports for a specific use category and nt represents the number of taxa used for that use category by all informants.

#### 2.4.2. Fidelity Level (FL)

This is used in ethnobotanical studies to assess the importance of a specific plant species for a particular community or culture. FL is calculated by determining the percentage of informants within a community who mention the use of a particular plant species for a specific ailment or purpose. A high FL indicates that the plant is consistently and commonly used for that particular purpose within the community, suggesting its potential effectiveness for that ailment or condition. FL value was calculated using the formula FL = Ip/Iu × 100, where Ip is the number of informants who reported the utilization of medicinal plants against a specific ailment and Iu is the total number of informants who mentioned the same plant against any ailment.

## 3. Result and Discussion

### 3.1. Major Crops Grown and Economic Activities in the Study Area

In the Ensaro district, the majority of the population, accounting for 93.34%, relies on farming as their main source of income. However, there is also a small percentage, about 4.74%, that engages in alternative income-generating activities. The district is involved in various agricultural practices such as the cultivation of cereals, vegetables, spices, root crops, and different fruits (Table 1). They also engage in irrigation and livestock production. According to the information obtained from the Ensaro district agricultural development office (Ewado, 2018), notable livestock numbers include 30,229 cattle, 9657 pack animals, 13,335 sheep, 14,261 goats, and 38,819 poultry.

### 3.2. Sociodemography of the Informants

In the study, 100 informants were chosen, with 80% being male and 20% female. Out of these informants, 43% were between the ages of 20 and 40, and the remaining 57% were above 40 years old. The majority of informants 58% were educated and the remaining 42% were illiterate and 98% informants' occupations were farmer (Table 2).

### 3.3. Taxonomic Diversity of Medicinal Plants in the Study Area

A total of 47 medicinal plant species were identified, distributed across 44 genera and 31 families. Among these, the families Solanaceae, Lamiaceae, and Asteraceae each contained five species. The families Fabaceae, Rutaceae, and Vitaceae included three, two, and two species, respectively. The remaining families were each represented by a single species (Table 3). Of the total medicinal plant species documented, 21 (44.68%) were used exclusively for treating livestock ailments, while the remaining 26 species (55.32%) were employed in the treatment of both human and livestock ailments (Table 4).

### 3.4. Growth Forms of Medicinal Plants

**Figure 2:**
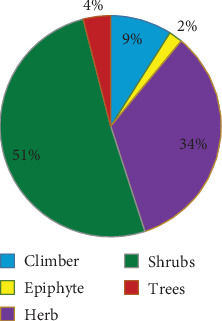
The growth form of medicinal plant.

According to the growth form analysis, the majority of the collected plant species fall under the category of shrubs, accounting for 51%. The herbaceous growth form comes next, representing 34% of the species. The remaining plant species are distributed among trees (4%), climbers (9%), and epiphytes (2%) (Figure 2).

### 3.5. Habitat of Medicinal Plants

**Figure 3:**
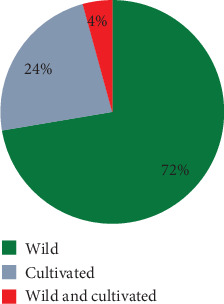
Habitat of medicinal plant.

Based on the data analysis, the majority of the medicinal plant species, accounting for 72%, are harvested from wild vegetation such as roadside areas, fencing, and rocky areas. Following this, 24% of the species are cultivated. The remaining plant species, amounting to 4%, are collected from either home gardens or farming lands (Figure 3).

### 3.6. Condition of Medicinal Plants

**Figure 4:**
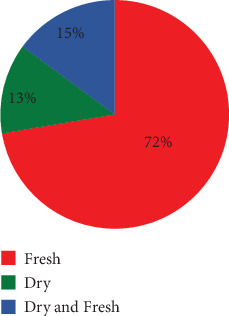
Condition of prepared medicine.

The local people of the study area prepared the remedies under different conditions (Figure 4). According to the information gathered from the informant, the local people reported that they prepared remedies using fresh, dried, or both types of plant materials. The result showed that the majority (72%) of medicinal plants were prepared in fresh conditions whereas 13% was prepared in dry conditions and the remaining (15%) was reported to be used in both dry and fresh forms.

### 3.7. Plant Part Used to Treat Livestock Disease

**Figure 5:**
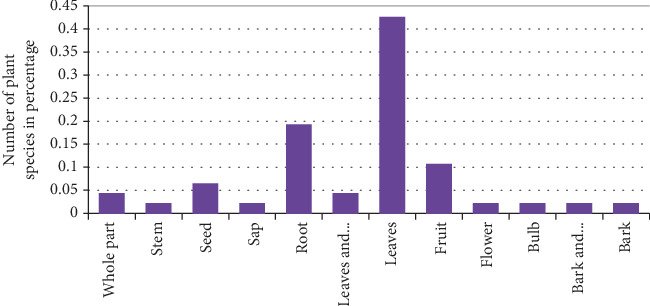
Percentage of plant parts used in the treatment of livestock aliment.

According to the analysis, leaves constitute the largest proportion of the plant parts used for preparing remedies for animal diseases, accounting for 42.55% of the total preparations. Roots take the second most commonly used plant part, accounting for 19.15% of the preparations. The remaining medicine is prepared from bark, stem, sap, fruit, flower, and the whole part of the plant (Figure 5).

### 3.8. Mode of Application Medicine

**Figure 6:**
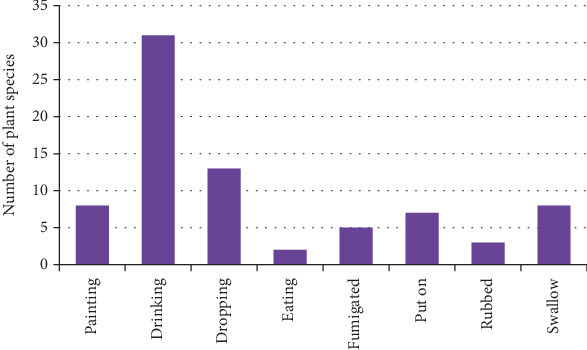
Percentage of the mode of application medicine to treat livestock disease.

Various modes of application are used to administer different medicines for the treatment of livestock diseases. The most common mode is through oral ingestion (drenching), accounting for 40% of the administrations, followed by topical application at 17%. Other modes include painting, ingestion, fumigation, rubbing, swallowing, and directly applying to the affected part (Figure 6).

### 3.9. Route of Administration

**Figure 7:**
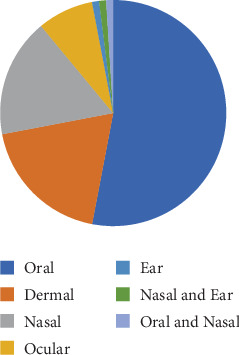
Percentage of route of administration of medicine to treat livestock disease.

Different routes of administration were used for applying local traditional medicines in the study area. The primary routes of administration included oral, dermal, nasal, ear, and ocular. Among these, oral administration was the most commonly used route, accounting for 53% of the cases. The dermal route was the second most prevalent, with a usage rate of 19%, followed closely by nasal administration at 17% (Figure 7).

### 3.10. ICF

ICF values for the different categories of disease were evaluated, and the result shows the ICF from the lowest 0.333 to the highest 1. The highest ICF disease categories were retained placenta (1.00) followed by anthrax (0.91), eye infection (0.90), diarrhea (0.89), rabies (0.88), leech infestation (0.808), and snake poisoning (0.75) (Table 5). The medicinal plant used to treat disease categories that have high ICF values were *Linum usitatissimum* used to remove (treat) retained placenta that caused the problem during giving birth; *Verbascum sinaiticum*, *Vernonia amygdalina*, *Capsicum annuum*, *Citrus urantifolia*, and *Cynodon dactylon* used to treat anthrax; *Solanum anguivi*, *Salvia nilotica*, *Permna shimperi*, *Euclea divinorum*, *Capparis tomentosa*, and *Asplenium aethiopicum* used to treat eye infection; *Linum usitatissimum*, *Lenses culenrise*, *Cucumis ficifolius*, and *Ajuga integrifolia* used to treat diarrhea; *Calpurnia aurea* and *Cyphostemma cyphopetalum* effective to treat rabies; and *Steganotaenia araliacea*, *Solanum incanum*, *Sideryoxylon oxyacanthum*, *Salvia nilotica*, *Nicotiana tabacum*, *Maytenus abutifolia*, *Grewia ferruginea*, *Cynoglossum* spp., *Cissus* spp., and *Buddleja polystachya* used to treat leech infestation.

### 3.11. FL

The top-ranking medicinal plants with a FL of 100% are *Sideryoxylom oxyacanthum* for leech infestation, *Inula confertiflora* for eye infection, and *Nicotiana tabacum* also for leech infestation. These plants are commonly used by the community to treat these specific ailments (Table 6).

## 4. Threats to Medicinal Plants

In the case of the threat factors mentioned by the informants, due to high population pressure, the expansion of agriculture and collection of firewood may disrupt the availability and access to the medicinal plants used in ethnoveterinary medicine. Consequently, this can limit the effectiveness of traditional treatments for animals and contribute to the decline of medicinal plant species used in such practices. To counter these threats, it is crucial to advocate for sustainable farming methods and educate people about the significance and preservation of these medicinal plants.

## 5. Discussion

Ethnoveterinary medicine offers a viable and cost-effective healthcare solution for animals, particularly in areas where traditional medicinal plants are more accessible and affordable than modern veterinary treatments. This approach holds great potential for providing sustainable and accessible healthcare options for animals in these communities. The present study revealed that the district has relatively high taxonomic diversity in ethnoveterinary medicinal plants with 47 species reported and which are found under 44 genera and 28 families. From the total of 28 families, Asteraceae represented 5 (11%), Solanaceae 5 (11%), Lamiaceae 5 (11%), Solanaceae represented 5(11%), and Fabaceae 3(6%) family Asteraceae finding the highest number of medicinal plants; this may be attributed to its dominance in terms of species diversity, abundance and wide distribution of members of the family in the flora of Ethiopia and Eritrea. Other studies conducted elsewhere in Ethiopia Asteraceae as the most dominant medicinal plant family [15, 16] and other studies which conducted in Pakistan by [17] revealed that most of the plants used for livestock ailment treatments belong to the family Solanaceae and Lamiaceae. In contrast to this study, other studies by ([18] and [19]) have reported that Fabaceae is the most dominant medicinal plant family.

Concerning the habitat of medicinal plants, the majority of these plant species (34 species, 72%) are harvested from wild vegetation like roadside, fencing, and rock. While 11 species (23%) were harvested from the home garden, the remaining (two species 4% collected from both wild and cultivated areas). Similar ethnobotanical studies conducted in different locations have demonstrated that natural habitats are crucial sources of medicinal plants [20, 21].

The traditional knowledge of medicinal plants is predominantly passed down through generations within families, often through oral means. This finding is consistent with the research conducted by [22], which similarly demonstrated that oral transmission from family members is a common source of healing wisdom. This study shows that the most dominant growth form analysis of medicinal plants in the study area showed that shrubs are the dominant life form of the medicinal plants represented by 51% followed by herbs at 34%. Similar studies have shown by [23–27] revealed that shrubs were the most used form of medicinal plants. However, some ethnoveterinary studies [8, 28–31] indicated that herbs were the most frequently used plant categories and other studies conducted in the Jimma zone by [32]; it was found that trees have predominantly been used for medicinal purposes, specifically for treating livestock ailments, compared to other types of plants. The present study shows that the local people use different plant parts to prepare the local remedies. According to the information gathered from informants for the remedy preparation, the most frequently utilized plant part is leaves (43%) followed by roots (19%). One possible reason for the greater preference for leaves over other parts of the plant is that leaves are easier to prepare and collect and tend to have higher concentrations of active ingredients that are beneficial for treating diseases. Much research has been conducted on different parts of Ethiopia consistent with this research finding. Ethnobotanical studies conducted by ([33] and [24]) reported that leaves were the most cited plant parts used in remedy preparations. Other studies on the contrary, research conducted by [8, 27, 28, 34–36] reported different results which showed that those roots were the most frequently utilized plant parts. When roots, barks, and the whole plant are harvested, it can disturb the growth and reproductive ability of the plants. As a result, certain plant species may become less abundant and face the risk of extinction. The people in the study area created medicine using both fresh and dried materials. While most remedies were made with fresh ingredients, some were prepared in dried form to ensure they could be used for a longer period, particularly for medicinal plants that were only available seasonally. This research finding is consistent with the findings of [33, 24].

People in the study area use different ways of administration based on the nature of remedies. The major routes of administration in the study area are oral, dermal, nasal, ear, and ocular way of administration. Oral administration is the dominant route (53%), followed by the dermal route (19%) applied by drinking and dropping, respectively. These results are consistent with the findings of various ethnobotanical research in different areas of Ethiopia. [37–39] reported that most medicinal plant remedies pastoralists were administered through oral.

The highest ICF disease category was retained placenta; Linum *usitatissimum* is the highest preferred medicinal plant by the local community to treat the retained placenta and other animal diseases. Several studies have shown that *Linum usitatissimum* contains important phytochemicals such as *ω*-3 fatty acids, phytoestrogenic-lignans (secoisolariciresinol diglucoside-SDG), phenols, flavonoids, sterols, proteins, and antioxidants, as well as various soluble and insoluble fibers. These compounds have been found to be beneficial in treating a variety of diseases including cancer, antioxidant-related conditions, antimicrobial infections, inflammatory disorders, obesity, diabetes, diarrhea, malaria, liver protection, kidney protection, immune system suppression, heart rhythm abnormalities, and cognitive health effects [40]. The plants *Sideryoxylom oxyacanthum*, *Inula confertiflora*, and *Nicotiana tabacum* showed the highest FL values in treating leech infestation and eye infection. Hence, medicinal plants with high IFC and FL values across various disease categories should undergo further phytochemical studies to explore their potential in effectively addressing the corresponding health conditions. A similar study conducted in another region of Ethiopia demonstrated that *Nicotiana tabacum* has significant potential as a medicinal plant for effectively treating leech infestation [41, 42].

Based on information obtained from an informant, several threat factors were identified, many of which are attributed to human activities. The rapid population growth has prompted the agricultural expansion and overharvesting of plants to make way for more farming land and the collection of firewood, which are considered the main threats to medicinal plants this research finding is in line with the research conducted in the Delanta and Ensaro district [43, 44]. Extensive research conducted in various regions of Ethiopia has yielded similar results, highlighting the strong correlation between high population growth and the endangerment of plant species used in traditional medicine. This study documented 47 medicinal plant species and their associated indigenous knowledge of the Ensaro district. From the total 47 medicinal plants, 21 medicinal plant species are used to treat eight different livestock aliments and 26 medicinal plants used to treat nine different human and livestock diseases. The majority of people in the study area still prefer to use local traditional medicines to treat different livestock diseases due to the lack of modern livestock clinics, unaffordable prices, and inadequate distribution in rural areas. The local community also utilized various animal-derived and other local materials to treat a range of animal and human ailments, in addition to their use of plant-based remedies.

## 6. Conclusion

Ensaro District is relatively rich in medicinal plant diversity and associated indigenous knowledge. Large numbers of medicinal plant species were collected from the wild whereas the remaining was collected from cultivated. The traditional healer reveals that leaves are frequently used plant parts followed by roots prepared mostly in fresh condition and mostly administered through the oral route, applied by drinking, eating, and swallowing. The highest FL values were obtained for the plants *Sideryoxylon oxyacanthum* treating leech infestation, *Inula confertiflora* used to treat eye infection and *Nicotiana tabacum* also for leech infestation. Therefore, further phytochemical investigations need to be made to the above-listed plants with the highest FL values which may indicate their higher potential against the respective ailments. Like most medicinal plants collected from wild habitats, therefore, awareness creation is the time needed to improve the local community's knowledge of the importance and management of plants both *in situ* and *ex situ*. Most of the plants are found under threats in the study area, which is directly related to the decline of traditional medicinal knowledge one of the main reasons for the decline of these traditional medicinal plants in the area arises from deforestation for firewood, charcoal, agricultural expansion, and construction. Conservation of these biological resources is very important, and the importance of maintaining knowledge about herbal medicine should be made among the healers to avoid erosion of the indigenous knowledge and to ensure its sustainable use.

## Figures and Tables

**Table 1:** Major crops grown in the study area (Ensaro District).

**Types of crops**	**Scientific name**	**Common name**	**Local name (Amharic)**
Cereal crops	*Triticum* spp. L.	Wheat	Sinde
	*Eragrostis tef* (Zucc.)	Teff	Tef
	*Sorghum bicolour* (L.) Moench	Sorghum	Mashela and zengada
	*Hordeum vulgare* (L.)	Barely	Gebese
	*Zea mays* (L.).	Maize	Bekolo

Pulse crops	*Pisum sativum* (L.)	Pea	Ater
	*Vicia faba* (L.).	Bean	Bakela
	*Lathyrus sativus* (L.).	Grass pea	Goaya
	*Cicer aertinum* (L.)	Chickpea	Shemebera
	*Phaseolus vulgaris* (L.)	Haricot bean	Boleka
	*Lens culunaris* (Medik.)	Lentil	Meser
	*Vigna radiat* (L.) R. Wilczek	Mung bean	Masho
	*Allium cepa* (L.).	Onion	Keyeshekurte

Vegetables	*Lactuca sativa* (L.)	Lettuce	Selata
	*Lycopersicon esculentum* (Mill)	Tomato	Timatim
	*Allium porrum* (L.)	Onion	Baroshekurte
	*Allium sativum* (L.).	Garlic	Nechshekurete
	*Brassica integrifolia* (H. West) Rupr.	Cabbage	Tekilgomen
	*Brassica oleracea* (L.).	Spinach	Gomen
	*Brassica nigra* (L.) W.D.J. Koch)	Mustard	Senafech
	*Sesamum indicum* (L.)	Selit	Selit

Oil crops	*Capsicum frutescens* (L.).	Chilli	Yabeshakariya
	*Brassica carinata* (A.Braun)	Mustard	Gomenzer
	*Carthamus tinctorius* (L.)	Safflower	Yabeshasuff
	*Guizotia abyssinica* (L.f.) Cass	Noog	Noog
	*Lepidium sativum* (L.).	Lepidum	Feto
	*Linum usitatissimum* (L.)	Linseed	Teleba
	*Capsicum annuum* (L.).	Chilli	Berebera

Fruits	*Carica papaya* (L.).	Papaya	Papaya
	*Persea americana* (Mill.)	Avocado	Avocado
	*Citrus sinensis* (L.) Osbeck.	Orange	Bertukan
	*Citrus aurantifolia* (Christm.) Swingle.	Lemon	Lomi
	*Mangifera indica* (L.).	Mango	Mango
	*Musa X paradisiacal* (L.)	Banana	Muse

Stimulant	*Catha edulis* (Vahl) Endl.	Chat	Chat
	*Coffee arabica* (L.).	Coffee	Buna

Spice	*Trachyspermum ammi* (L.) Sprague	White cumin	Nech azemude
	*Nigella sativa* (L.).	Black clumin	Tekur azemud
	*Trigonella foenum-graecum* (L.)	Fenugreek	Abesh
	*Ocimum lamiifolum* Hochest ex Benth.	Black clumin	Besobila

Root crops	*Beta vulgaris* (L.).	Beetroot	Keye sere
	*Solanum tuberosum* (L.).	Potato	Dinich

*Note*: source of data: Ensaro Woreda Agricultural Development Office.

**Table 2:** Informant information.

**Demographic characteristics**									
**Age**	**Gender**	**No.**	**Education level**	**No.**	**Occupation**	**No.**	**Ethnic group**	**No.**	**Total**
20–40	Female	13	Illiterate	4	Farmer	12	Amhara	13	43
			Educated	9	Merchant	1			
	Male	30	Illiterate	9	Farmer	29	Amhara	30	
			Educated	21	Government	1			
>40	Female	6	Illiterate	4	Farmer	6	Amhara	6	57
			Educated	2					
	Male	51	Illiterate	25	Farmer	51	Amhara	51	
			Educated	26					

**Table 3:** Medicinal plant diversity within their family.

**No.**	**Family name**	**Frequency**	**Percentage**
1	Anacardiaceae	1	2.13
2	Apiaceae	1	2.13
3	Apocynaceae	1	2.13
4	Asclepiadoideae	1	2.13
5	Aspleniaceae	1	2.13
6	Asteraceae	5	10.64
7	Boraginaceae	1	2.13
8	Brassicaceae	1	2.13
9	Cappariaceae	1	2.13
10	Celastraceae	1	2.13
11	Cucurbitaceae	1	2.13
12	Ebenaceae	1	2.13
13	Fabaceae	3	6.38
14	Lamiaceae	5	10.64
15	Liliaceae (Alliaceae)	1	2.13
16	Linaceae	1	2.13
17	Loganiaceace	1	2.13
18	Lorathaceae	1	2.13
19	Myrtaceae	1	2.13
20	Olacaceae	1	2.13
21	Poaceae	1	2.13
22	Polygonaceae	1	2.13
23	Rhamanceae	1	2.13
24	Rutaceae	2	2.13
25	Sapnidaceae	1	2.13
26	Sapotaceae	1	2.13
27	Scrophulariaceae	1	2.13
28	Solanaceae	5	10.64
29	Tiliaceae	1	2.13
30	Vitaceae	2	4.265
31	Zingiberaceae	1	2.13
	Total	47	100%

**Table 4:** Ethnoveterinary plant species used for treating animal ailments in the study site.

**Scientific name/family name and voucher number**	**Local name**	**Habit**	**Habitat**	**Part used**	**Condition**	**Preparation**	**Mode of application**	**Method of preparation**	**Route**	**Disease treated**
*Ajuga integrifolia* Buch-Ham ex-D. Don. Lamiaceae MIK-001	Aremangusa	He	W	R	F	Leaves are crushed and given with water.	Drinking	Pounding	Oral	Diarrhea for both animal and human

*Allium sativum* L. Liliaceae MIK-002	Netch Shinkurt	He	Cu	Bulb	F	Crushed the mixture of *Allium sativum*, *Ruta chalepensis* lemon, honey, and *Citrus urantifolia* and finally drank it. Or soak the mixture of *Allium sativum*, *capsicum* spp., and lemon overnight and finally drunk.	Drinking	Pounding	Oral	Malaria

*Allophylus abyssinicus* Radlk. Sapnidaceae MIK-003	Enbuse	T	W	L	F	Pounded the leaves and drenched.	Dropping	Pounding	Nasal	Furo (nose disease)

*Asplenium aethiopicum* (Burm.f.) Bech Aspleniaceae MIK-004	Yedegayelebese	He	W	Hp	F	Powdered the leaves and applied them to the affected part.	Put on	Pounding	Ocular	Eye

*Buddleja polystachya.* Fresen. Loganiaceace MIK-005	Anfar	Sh	W	Bark L	F	Chewing the bark and swallowing.	Swallow	Chewing	Oral	Snakebite as first aid
						Chewing the leaves and drenching through the nose.	Dropping	Chewing	Nasal	Leech
						Pounded the mixture of leaves with *Rumex nervosus* together.	Drinking	Pounding	Oral	Furo (nose disease)

*Calotropis procera* (Aiton) Dryand. Apocynaceae sub family of Asclepiadoideae MIK-006	Kinbo	Sh	W	Sap	F	Pounded the leaves and mixed with water finally drenching.	Drinking	Pounding	Oral	Atabeko animal
						Chewing the root and swallow.	Swallow	Chewing	Oral	Snake poison
						Latex of *Calotropis procera* applies to it.	Dropping	Unprocessed	Dermal	Hemorrhoids

*Calpurnia aurea (*Ait). Benth. Fabaceae MIK-007	Degeta	Sh	W	L	D & F	Pound fresh leaves and mix with water drunk.	Drinking	Pounding	Oral	Jaundice
						The ground dried seed of *Calpurnia aurea* is mixed with dried powder leaves of *Ximenia Americana* and finally mixed with honey.	Eating	Powdering	Oral	Malaria, rabies, and stomach aches for both human and animal

*Capparis tomentosa* Lam.Capparaceae MIK-008	Gumero	Sh	W	L &R	F	The leaves chewing and applying to the affected eye.	Dropping	Chewing	Ocular	Eye disease and evil eye

*Capsicum annuum* L. Solanaceae MIK-009	Karia	He	Cu	Fr	D	Pounded the mixture of *Capsicum annuum* L, *Allium sativum*, *Ruta chalepensis*, and *Eucalyptus globules* then drenching.	Drinking	Pounding	Oral	Abasenga/Anthrax/

*Carissa spinarum* L. Apocynaceae MIK-010	Agam	Sh	W	R	D& F	The root of *Capparis tomentosa* mixed with *Carissa spinarum* dried and smoked.	Fumigating	Smoking	Nasal	Evil eye
						Chewing seven fresh leaves and swallowing.	Swallow	Chewing	Oral	Snake poison

*Chrysanthemum indicum* (L.) Asteraceae MIK-011	Fafugn	He	W	L	F	Pound the leaf and mix it with the Vaseline applied affected skin part.	Brushing	Pounding	Dermal	Jaundice in both H & A, hemorrhoid

*Cissus* spp. Gilbert. Vitaceae MIK-012	Ye Oromo ekoye	Cl	W	L	F	Chopped and pounded the leaves and drenching.	Drinking	Pounding	Oral	Leech

*Citrus urantifolia (*Christm.) wingle. Rutaceae MIK-013	Lomi	Sh	Cu	Fr	F	The lemon squeezes and coats on affected body part.	Painting	Squeeze	Dermal	Skin itch
						Pounded the mixture of *Ruta chalepensis* leaves, *Allium sativum*, lemon juice, and areka and drunk.	Drinking	Pounding	Oral	Abasenga/Anthrax/

*Cucumis ficifolius* A. Rich. Cucurbitaceae MIK-014	Yemeder embuye	Cl	W	R.	F	Chewing the root and swallow.	Swallow	Chewing	Oral	Stomach ache
						Cutting the root by calling the name of the affected person and the disease.	No	Cutting		Diarrhea
						Chewing the root and swallow.	Swallow	Chewing	Oral	Snakebite

*Cynodon dactylon* (L.) Pers. Poaceae MIK-015	Serdo	He	W	Wp	F	Crushed and tied on the affected part.	Put on	Pounding	Dermal	Wound and Abasenga

*Cynoglossum* spp. Boraginaceae MIK-016	Chemed Ketel	Cl	W	L	F	Pounded the fresh leaves with the leaves of *Zehneria scabra* and applied them to the wound.	Painting	Pounding	Dermal	Yegedegedewa
						Pounded the leaves and drenched with a left nose.	Dropping	Pounding	Nasal	Leech

*Cyphostemma cyphopetalum* (Fresen.) Vitaceae MIK-017	Gindosh	Cl	W	Stem	D & F	Chewing the stem with honey.	Swallow	Chewing	Oral	Snakebite
						Crushed the stem and tied it to the affected part.	Put on	Crushing	Dermal	Wound
						Peeling the stem, chop it up, and dry and ground, then the powder mix with a small amount of water and drink it.	Drinking	Powdering	Oral	Rabies for both human and animal

*Echinops kebericho* Mesfin. Asteraceae MIK-018	Kebercho	Sh	W	R	D	Smoke kebercho	Fumigating	Smoking	Nasal	Furo (nose disease of animal
						Smoke kebercho	Fumigating	Smoking	Nasal	Evil eye

*Eucalyptus globules* (Labill.) Myrtaceae MIK-019	Nech bahirzaf	T	C	L	F	Chopped, boiled, and inhale the vapor.	Fumigating	Boiling	Nasal	Mich
						Mix pounded leaves of *Eucalyptus globulus*, *Ocimu mlamiifolium*, and *Croton macrostachyus*, then boiled and fumigate.	Fumigating	Boiling	Nasal	Headache and mitch

*Euclea divinorum* Hiern. Ebenaceae MIK-020	Dedeho	Sh	W	R	F	The root is chewed and applied to the eye.	Put on	Chewing	Ocular	Eye and evil eye

*Grewia ferruginea* Hochst. ex A. Rich. Tiliaceae MIK-021	Lenequta	Sh	W	Bark	F	Pounded the bark and mixed with boiled linseed then drenched.	Drinking	Pounding	Oral	Leech
						Pounded the bark and mixed with chewed *Vicia faba*.	Put on	Pounding	Dermal	Ebach

*Haplocarphas chimperi* (Sch. Bip.) Beauv. Asteraceae MIK-022	Nech Ketel	He	W	R	F	The root is crushed and applied to the affected boy part.	Put on	Crushing	Dermal	Wound (ebach) for animal

*Helixanthera* spp. Lorathaceae MIK-023	Teketsela	Epi	W	L	D	Crushed, dried, and powdered and applied on the affected part.	Painting	Powdering	Dermal	The wound on the donkey's back

*Lens culenris* Medik. Fabaceae MIK-024	Mesir	He	Cu	Seed	D	The seed of *Lenses culenrise* is mixed with *Allium sativum*, and *Capsicum annuum* is pounded and eaten with injera.	Eating	Pounding	Oral	Diarrhea sheep, goat
						Chewing the seven seeds and apply the affected part.	Painting	Chewing	Dermal	Skin disease caused by spider

*Lepidium sativum* L Brassicaceae. MIK-025	Fetto	He	Cu	Seed	D	Pounded the seed and tied the affected body part.	Painting	Pounding	Dermal	Skin allergy caused by spiders (Yegedegedewa)

*Linum usitatissimum* Linaceae MIK-026	Teliba	He	Cu	S	D	Ground the seed very fine and boil it with water then drink it.	Drinking	Powdering	Oral	Undersized calf
						Boiled the seed with sugar and drunk.	Drinking	Boiling	Oral	Broken bone and back pane
						Boiled with water and drunk.	Drinking	Boiling	Oral	Diarrhea and remove placenta

*Maytenus arbutifolia* (A.Rich.) Wilczek.Celastraceae MIK-027	Atate	Sh	W	L	F	Pound the leaves and then drink it for the animal.	Drinking	Pounding	Oral	Leech

*Nicotiana tabacum* L. Solanaceae. MIK-028	Tenebaho	Sh	Cu	L	F	Crushing the leaves and drenching with the left nose.	Dropping	Pounding	Nasal	Leech

*Permna shimperi* Engle. Lamiaceae MIK-029	Chocho	Sh	W	L	D & F	Pound the leaf and apply it to the affected eye part or use *Inula confertiflora* (woyenagift) leaf.	Dropping	Pounding	Optical	Eye disease for animal
						Mix the ash of burned wood with water.	Painting	Pounding	Dermal	Animal wound
						Pound the leaf and drunk to animal.	Drinking	Pounding	Oral	Jaundice

*Plectranthus* spp. Lamiaceae MIK-030	Dachet	He	W	L	F	Pounded the leaves and mixed with water finally drenching them.	Drinking	Pounding	Oral & nasal	Leech

*Rhamnus prinoides* L*'*Herit. Rhamanceae MIK-031	Gesho	Sh	Cu	L	F	Pounded the leaves and mixed with water then drenched them.	Drinking	Pounding	Oral	Atabeko
						Chewing the leaves swallow.	Swallow	Chewing	Oral	Snake poison

*Rhus natalensis* Krauss. Anacardiaceae MIK-032	Takema	Sh	W	L	F	Pound the leaves and drenched.	Drinking	Pounding	Oral	Atabeko

*Rumex nervosus*Vahl. Polygonaceae MIK-033	Enbuacho	Sh	W	Flower	F	Pound the flower and mix water drenching through the nose.	Dropping	Pounding	Nasal	Furo (nose disease)

*Ruta chalepensis* L. Rutaceae MIK-034	Tenadam	Sh	Cu	L	F	Pounded The mixture of *Ruta chalepensis* leaves, *Allium statium*, lemon juice, and areka and drunk.	Drinking	Pounding	Oral	Stomach ache

*Salvia nilotica* Juss. ExJacq. Lamiaceae MIK-035	Hulegeb	He	W	L	F	Pound the leaves and drunk.	Drinking	Pounding	Oral	Tonsil
						Pound the leaves and apply them to the affected part.	Put on	Pounding	Ocular	Eye animal
						Pound the leaves and apply them to the affected part.	Painting	Pounding	Ear	Ear disease animal
						Pounded the leaves mixed in water and drunk.	Drinking	Pounding	Oral	Leech
				R		The root is crushed and given water.	Drinking	Crushing	Oral	Headache

*Senna singueana* (Del.) Fabaceae MIK-036	Gufa	He	W	R	D & F	Smashed the root and swallow	Swallow	Pounding	Oral	Snake poison
						Rubbed affected parts with fresh leaves.	Rubbing	Unprocessed	Dermal	Itching animal skin
						Dry and powder the mixture of *Senna* roots, and root of *Caparise tomantosia* and finally mix with Vaseline.	Painting	Powdering		Begungi

*Sideroxylon oxyacanthum.* Baill Sapotaceae. MIK-037	Dameza	Sh	W	L	F	Pounded the leaves and mixed with water finally drenching them.	Dropping	Pounding	Nasal	Leech

*Solanecio gigas* (Vatke) C. Jeffrey. Asteraceae MIK-038	Yeshekoko gomen	Sh	W &Cu	L	F	Crushed, squeezed, and rubbed on affected parts.	Rubbing	Pounding	Dermal	Jaundice (which occurs on skin, itching)

*Solanum anguivi* Lam. Asteraceae MIK-039	Zerichmembuaye	Sh	W	Fr	F	Cut the fruit in half and apply it to the affected part.	Dropping	Unprocessed	Ocular	Eye

*Solanum incanum* L. Solanaceae MIK-040	Embuay	Sh	W	Fr	F	Pounded the mixture of dameza leaves and fruit of *Solanum incanum* then dropped it in the left nose.	Dropping	Pounding	Nasal	Leech

*Solanum marginatum* L.f. Solanaceae MIK-041	Geber embuay	Sh	W	Fr	F	Cut the fruit and rub the affected part.	Rubbing	Unprocessed	Dermal	Ruret (skin disease)

*Steganotaenia araliacea* Hochst. Apiaceae MIK-042	Yejib merkus	Sh	W	L	F	Pounded fresh leaves and mixed with water and drank through the nose.	Dropping	Pounding	Nasal	Leech

*Thymus schimperi* Ronniger. Lamiaceae MIK-043	Tosign	He	W	L	D & F	Mix the Powdered leaves with water then boiled finally drinks it.	Drinking	Powdering	Oral	Cough
						Pounded the mixture of *Thymus schimperi* leave, *Eucalyptus globulus*, and *Cucumis ficifolius.*	Drinking	Pounding	Oral	Mognbagegn

*Verbascum sinaiticum* Benth. Scrophulariaceae MIK-044	Yaheya joro	He	W	R		Pound the root, leaves of *Foeniculum vulgare* and *Eucalyptus globules* then drunk it.	Drinking	Pounding	Oral	Aba senga
					F	The pounded leaves drenched through the left nose and left ear.	Dropping	Pounding	Nasal and ear	Abasega

*Vernonia amygdalina* Del. Asteraceae MIK-045	Grawa	Sh	W & Cu	L	F	Pound the mixture of leaves of *Cynodon dactylon* and *Eucalyptus globules* and drenched.	Drinking	Pounding	Oral	Abasega
						Pounded the leaves with water and drank the filtrate.	Drinking	Pounding	Oral	Stomach ache

*Ximenia Americana* L. Olacaceae MIK-046	Enkoye	Sh	W	L	D & F	Pound fresh leaves and swallow	Swallow	Pounding	Oral	Jaundice
						Mix the dried grounded leaves of *Ximenia americana* and *Calpurnia aurea* seed, finally drenching with water.	Drinking	Pounding	Oral	Goleba and undersized livestock

*Zingiber officinale* Roscoe. Zingiberaceae MIK-047	Zengebel	He	Cu	R	F	Crushed with water and drank.	Drinking	Pounding	Oral	Cough
						Pound the mixture of *Lepidium sativum* seed, *Ruta chalepensis* leaves, and *Zingiber officinale* and drink with butter.	Drinking	Pounding	Oral	Stomach ache

Abbreviations: Ba, bark; climbe, r; Cu, cultivated; drie, d; Fr, fruit; fres, h; her, b; lea, f; roo, t; Se, seed; Sh, shrub; St, stem; tre, e; wil, d; WP, whole.

**Table 5:** The informant consensus factor (ICF) values for the various disease categories in the study district.

**Animal disease categories**	**Number of plant species used**	**Number of use reports**	**ICF**
Retained placenta	1	5	1.00
Anthrax (abasenega)	5	9	0.91
Eye infection	6	51	0.90
Diarrhea	4	30	0.89
Rabies	2	10	0.88
Leech infestation	10	48	0.80
Snake poisoning	6	18	0.75
Bloat	14	38	0.64
Wounds (broken bone and nose disease)	8	16	0.53
Skin disease	13	25	0.50
Jaundice	3	4	0.33

**Table 6:** Fidelity level of highly utilized species.

**No.**	**Plant name**	**Ailment category**	**IU**	**IP**	**FL value (100%)**
1	*Sideroxylon oxyacanthum*	Leech infestation	12	12	100
2	*Inula confertiflora*	Eye infection	9	9	100
3	*Nicotiana tabacum*	Leech infestation	16	14	87.5
4	*Permnashimperi*	Eye infection	14	12	85.7
5	*Rumex nervosus*	Wound healing	10	8	80
6	*Calpurnia aurea*	Skin infection	5	4	80
7	*Solanecio gigas*	Jaundice	3	2	66.66
8	*Linum usitatissimum*	Retained placenta	5	2	40
9	*Cucumis ficifolius*	Blackleg	41	15	36.58
10	*Cucumis ficifolius*	Anthrax	41	7	17

## Data Availability

The data used to support the findings of this study are included in the article.

## Supporting Information

Supporting Information Additional supporting information can be found online in the Supporting Information section. Checklist of questions used to collect ethnoveterinary botanical data. Checklist of questions used to collect ethnoveterinary botanical data.
