# Supplementary material for: The Ethnoveterinary Study of Medicinal Plants Utilized in Treating Animal Diseases in Ensaro District, North Shewa Zone of Amhara Regional State in Ethiopia
Source: ScientificWorldJournal. 2025 Jul 9;2025:3038829. doi: 10.1155/tswj/3038829 (PMC12267971; doi:10.1155/tswj/3038829)
Supplement: Supporting Information — Additional supporting information can be found online in the Supporting Information section. Checklist of questions used to collect ethnoveterinary botanical data. Checklist of questions used to collect ethnoveterinary botanical data. [file 3038829.f1.docx]

**Checklist of questions used to collect ethno-veterinary botanical data.**

Informants’ consent for the participation in the study:

I..................................................... (name of informant) hereby give my full consent and conscious to participate in this study and declare that to the best of my knowledge the information that I have provided are true, accurate and complete.

Date................ signature ........................

**General information on the respondents**

Name

Gender

Age

Occupation

Education level

Location/Residence

Religion

Ethnicity

**Ethno-veterinary Information**

What are the most common diseases of animal in your area?

What action takes to against the above disease?

List plant species used to treat livestock in your area?

Local name of the plant

Plant part used

Plant habit

Habitant of the plant

Preparation of the medicine

How to apply

Route of administration
